# Radiotherapy results in decreased time to second cancer in children with Li Fraumeni syndrome

**DOI:** 10.1093/jnci/djaf057

**Published:** 2025-03-10

**Authors:** Emma R Woodward, John-Paul Kilday, Stephanie Ng, Anna Kelsey, D Gareth R Evans

**Affiliations:** Division of Evolution, Infection and Genomic Sciences, Faculty of Biology, Medicine and Health, University of Manchester, Manchester, United Kingdom; Division of Cancer Sciences, Faculty of Biology, Medicine and Health, University of Manchester, Manchester, United Kingdom; Manchester Centre for Genomic Medicine, Manchester University NHS Foundation Trust, Manchester, United Kingdom; Royal Manchester Children’s Hospital, Manchester University NHS Foundation Trust, Manchester, United Kingdom; Division of Cancer Sciences, Faculty of Biology, Medicine and Health, University of Manchester, Manchester, United Kingdom; Royal Manchester Children’s Hospital, Manchester University NHS Foundation Trust, Manchester, United Kingdom; Division of Evolution, Infection and Genomic Sciences, Faculty of Biology, Medicine and Health, University of Manchester, Manchester, United Kingdom; Division of Cancer Sciences, Faculty of Biology, Medicine and Health, University of Manchester, Manchester, United Kingdom; Manchester Centre for Genomic Medicine, Manchester University NHS Foundation Trust, Manchester, United Kingdom

## Abstract

Li Fraumeni syndrome (LFS) arising from germline *TP53* mutation results in defective DNA repair and increased risk of multiple primary cancers beginning in childhood. Curative intent radiotherapy is often used to treat childhood cancer, but its impact on children with LFS has not been reviewed.

We undertook a retrospective case-series review of 47 children with a solid cancer diagnosed age less than 16 years to assess time and survival after second cancer diagnosis.

After radiotherapy for the first cancer diagnosis, median time to second primary cancer diagnosis was 13.3 years and median survival 9.7 years. Where no radiotherapy was received, median time to second primary cancer diagnosis was 25.1 years (χ^2^ = 14.8, *P* < .0001; Hazard Ratio = 7.9 [95% CI = 2.8 to 22.6]), and median survival of 29.2 years (χ^2^ = 12.5, *P* = .004, Hazard Ratio = 3.2 [95% CI = 1.5 to 6.6]).

Radiotherapy for first cancer in children with LFS is associated with adverse outcomes and ought to be considered only in the absence of other potentially curative options. Where unavoidable, second cancer risks must be minimized.

Li Fraumeni syndrome (LFS) is a high-risk age-related cancer predisposition syndrome arising from (likely) pathogenic variants (PGVs) of *TP53.*^1^ This risk begins from birth with 20% of children developing a cancer by age 5 years, 40% by age 18 years, and greater than 40% of individuals will develop multiple metachronous primary tumors.^2^ The classical cancer spectrum includes adrenocortical carcinoma (ACC) in children, osteo- and soft tissue sarcoma (OS, STS) and central nervous system tumors in adults and children, and breast cancer in women.^1^^,^^2^

P53 plays a critical role in DNA repair. In p53-deficient cells, DNA damage acquired through ionizing radiation risks being propagated, resulting in increased risk of neoplastic transformation. In view of this, recent guidance suggests that, for LFS, where a cancer has developed the use of curative intent radiotherapy (XRT) should be limited where possible.^3^^,^^4^ This is despite a lack of direct evidence of an increased risk of second primary malignancies beyond anecdotal or small reports.^5^ Given this guidance, the challenges of managing childhood cancers occurring in LFS in view of the remaining years of life at risk from further primary cancers and the lack of direct evidence, we undertook a retrospective evaluation of the outcomes after radiotherapy for LFS-associated childhood cancers in our institution to attain data to help inform management.

In this service evaluation study, we interrogated the Manchester Children’s Tumour Registry data from the Royal Manchester Children’s Hospital (1954-2012)^6^ and the Manchester Centre for Genomic Medicine (MCGM) (1990-present) for information regarding radiotherapy treatment in children with solid malignancies from families with LFS. Germline *TP53* pathogenic variants were identified from blood or stored tissue block material as previously described.^7^ Children with hematological malignancy were excluded. Statistical analysis was undertaken using GraphPad Prism version 10.2.0 for Windows (GraphPad Software, San Diego, CA, USA). A *P* value less than .05 was considered statistically significant.

We identified 47 children from 40 LFS families (34 families—1 child, 6 families—2 children) with one or more cancer diagnosis occurring at age less than 16 years (age at first cancer diagnosis: mean 5.0 years [95% CI = 3.6 years to 6.4 years], range 0-15 years, SEM = 0.7, median = 3 years, 25^th^ percentile = 1 year, 75^th^ percentile = 7 years) from December 1, 1947 (1947 case identified from second cancer in 1954) to May 13, 2021. In total, 21/47 had ≥2 cancer diagnoses, and 10 of these had ≥3 cancer diagnoses during their lifetime/follow-up.

In total, 22 children received XRT treatment (start date ranges: December 1, 1947 to June 7, 2021) as treatment for the first cancer, and 25 children did not (Table S1).

Median time to second primary cancer after the first cancer primary diagnosis was 13.3 years in children receiving XRT for the first cancer compared with 25.1 years for those who did not (χ^2^ = 14.8, *P* < .0001; Hazard Ratio (HR) = 7.9 [95% CI = 2.8 to 22.6]) (Figure 1, A). Median age at second primary cancer was 18.7 years for those children receiving XRT for their first cancer compared with a median age of 28.0 years for those not receiving XRT (χ^2^ = 14.3, *P* = .0002; HR = 7.7 [95% CI = 2.7 to 22.0]) (Figure 1, B). Time to third primary cancer where XRT was received for the first primary cancer was also significantly reduced (χ^2^ = 15.0, *P* = .0001; HR = 10.9 [95% CI = 2.4 to 50.2]). Our data were too few on those receiving XRT for the second cancer to analyze.

**Figure 1.**
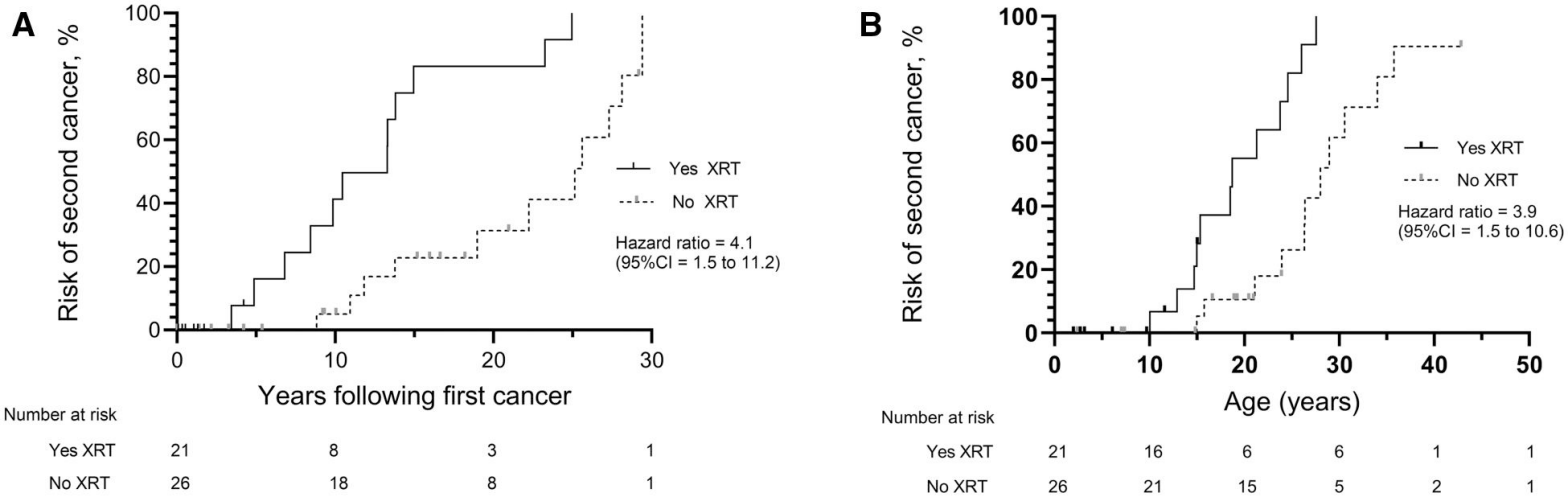
Time to second primary cancer (**A**) and age at second primary cancer (**B**) with or without radiotherapy (XRT) for the first primary cancer.

As MCGM records commenced in 1990, we also undertook analysis of time to second and third primary for first primary cancer occurring in 1990 or later. Again, this showed significantly reduced time to second (XRT/no XRT for first cancer, median time to second cancer = 9.5/25.1 years, χ^2^ = 5.6, *P* = .0028; HR = 5.6 [95% CI = 0.8 to 40.6]) and third primary cancer diagnosis (χ^2^ = 9.9, *P* = .0017; HR = 73.6 [95% CI = 5.1 to 1074]).

There was no difference in age at first cancer diagnosis for those children receiving XRT for this cancer compared with those not (mean age first cancer diagnosis = 4.8 years XRT, 5.3 years no XRT, *P* = .7100).

Because ACC and OS/STS are core childhood cancers in LFS, we considered time to second primary cancer and survival from the first primary for these cancers. Adrenocortical carcinoma was associated with significantly decreased survival (Figure 2, A), but not time to second primary cancer, where XRT was used (median time to second primary: XRT = 13.8 years, no XRT = 25.1 years, χ^2^ = 3.1, *P* = .0807, HR = 2.6 [95% CI = 0.7 to 10.2]; median survival: XRT = 16.0 years, no XRT = 31.7 years, χ^2^ = 7.2, *P* = .0072, HR = 4.3 [95% CI = 1.2 to 15.9]). OS/STS was associated with both statistically significantly decreased time to second primary cancer, and survival (Figure 2, B), where XRT was used (median time to second primary: XRT = 10.5 years, no XRT = 29.4 years, χ^2^ = 8.2, *P* = .0041, HR = 6.0 [95% CI = 1.1 to 31.9]; median survival: XRT = 4.2 years, no XRT = 16.6 years, χ^2^ = 4.0, *P* = .0472, HR = 2.5 [95% CI = 0.9 to 6.8]).

**Figure 2.**
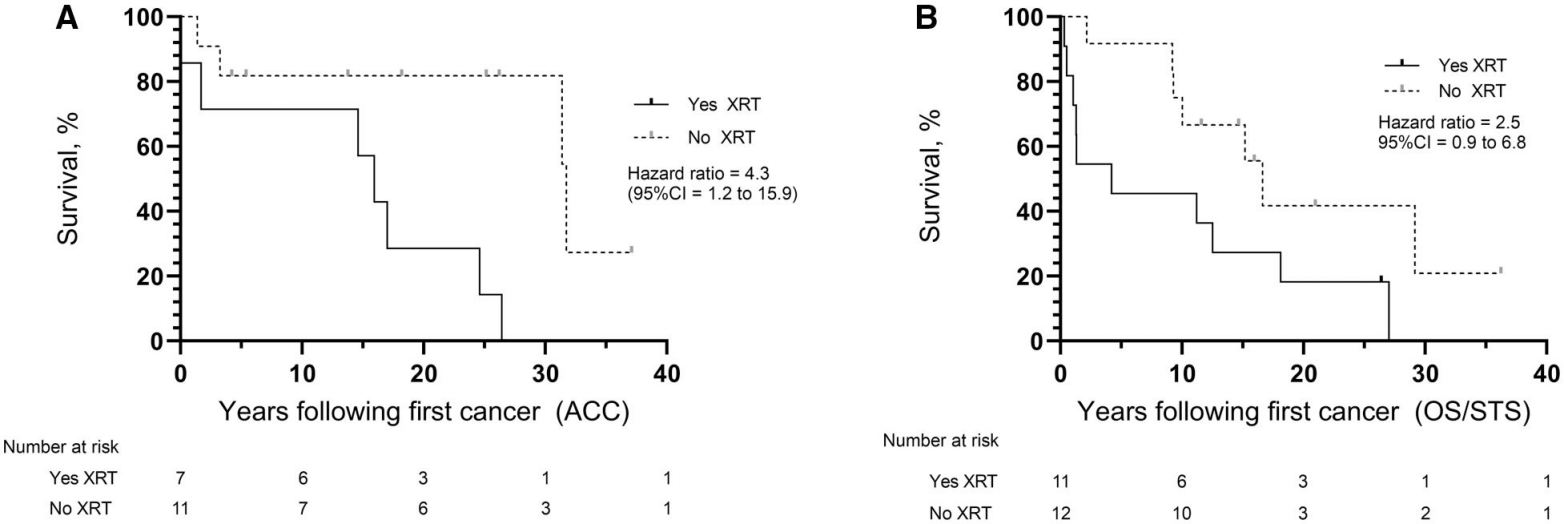
Survival with or without XRT to treat the first primary cancer. Survival following, as first primary cancer: adrenocortical carcinoma (ACC) (**A**); soft-tissue sarcoma/osteosarcoma (OS/STS) (**B**).

Nine children received XRT for the first primary cancer and subsequently developed a second primary cancer. All 9 second primary cancers were OS/STS, and all developed in the radiation field (*P* < .0001). Second cancers were new primary cancers and not recurrences of the first cancer as indicated by different histology (Table S1).

Overall survival after the first cancer diagnosis was significantly shorter where XRT was received for the first cancer compared to when XRT was not received (XRT-median survival 9.7 years; no XRT-median survival 29.2 years; χ^2^ = 12.5, *P* = .0004, HR = 3.2 [95% CI = 1.5 to 6.6]) (Figure 2, A). Survival after second primary cancer diagnosis was not significantly reduced where XRT was used as treatment for the first primary cancer (XRT median survival = 4.1 years; no XRT median survival = 8.5 years, χ^2^ = 3.6, *P* = .0571, HR = 3.3 [95% CI = 1.1 to 9.6]).

Considering first cancer diagnoses from 1990 onward (when MCGM records began) only, survival after the first primary cancer was also significantly reduced where XRT was used as treatment (XRT median survival = 5.5 years; no XRT median survival = 29.2 years, χ^2^ = 4.7, *P* = .0302, HR = 3.0 [95% CI = 0.9 to 10.2]).

We present a comprehensive analysis exclusively in children with LFS (germline *TP53*) related cancers. To our knowledge, it is the largest single dataset pertaining to children, and we detail outcomes for 47 children with LFS childhood solid cancer with or without curative intent XRT for the first cancer.

Median time to second tumor where XRT was received was 13.3 years, similar to that reported in other series of adults and children combined.^2^ Median survival for ACC and OS/STS (core LFS childhood cancers) was also significantly reduced where XRT was received. Similarly, significantly poorer outcomes have been previously noted after XRT in a much smaller series of 31 (4 children) individuals with LFS,^8^ although all the second cancers in our series were new primaries rather than recurrences.

This clinical dataset, and that of others,^4^ does suggest an inherent radiation resistance to treatment,^9^ and radiosensitivity for new malignancy,^10^ associated with defective p53. Considering a possible underlying cellular mechanism for this radiosensitivity, cells with dysfunctional p53 that have survived ionizing radiation prevent ATM shuttling to the nucleus, where it senses and amplifies the signal from dsDNA breaks to facilitate the repair pathway.^4^ Accumulation of these dsDNA repair breaks can then contribute to error propagation and cellular transformation. Of note, a similar mechanism has been shown for hereditary retinoblastoma, also associated with radiosensitivity.^4^^,^^11^

We acknowledge there are limitations with our study. Our data are drawn from a historical single-institution dataset. Although this results in an inherent bias, currently it is the only means of attaining data; in the future, more bias-free, agnostic datasets will be achieved through large-scale data-linkage studies—for example, the Cancer Research UK Data for Children’s and Young People’s Cancer initiative^12^ and initiatives for germline *BRCA1*/*2.*^13^ National data-linkage studies will also help ascertain whether all *TP53* PGVs are equally radiosensitive—for example, the classical dominant negative PGVs affecting codons 245/248, and the childhood cancer predominant substitutions, R337H and P152L^14^^,^^15^—and whether all cancer type outcomes are XRT are similar; we note the reported increased survival reported after XRT for LFS-associated medulloblastoma.^16^

We cannot exclude that poorer survival after XRT might be explained by prognostically unfavorable cancers being more likely to receive XRT—for example, tumor size not being amenable to curative surgery or limited medical treatment options for historical case patients. Certain tumor types requiring XRT may also imply a higher probability of a shorter sojourn time to second malignancy, but this does not explain the shortened time (although not significant) to second tumor after XRT for adrenal tumors.^14^

The management of cancers arising in children and young people with hereditary predisposition is challenging given the increased risk of metachronous cancers, particularly in LFS where this risk is greater than 40%. Although management of the first cancer typically takes precedence, life-long cancer risk and cancer specific mortality must be considered.

The shortened time to metachronous primary cancer we demonstrate does provide clinical evidence for current guidance that XRT should be avoided in children with LFS^3^ where possible. Although not conclusively demonstrated, our data do suggest that the shortened time may affect survival through causing the subsequent primary cancer earlier. Therefore, where no other potentially curative therapeutic options are available, then second cancer risk needs to be minimized—for example, using proton-beam approaches.^4^

## Supplementary Material

djaf057_Supplementary_Data

## Data Availability

This work uses data provided by patients and collected by the NHS as part of their care and support. The patients did not give written consent for their data to be shared publicly, so due to the sensitive nature of the research, supporting data are not available.
