# Supplementary material for: Radiotherapy results in decreased time to second cancer in children with Li Fraumeni syndrome
Source: J Natl Cancer Inst. 2025 Mar 10;117(10):2120–3. doi: 10.1093/jnci/djaf057 (PMC12505129; doi:10.1093/jnci/djaf057)
Supplement: djaf057_Supplementary_Data [file djaf057_supplementary_data.pdf]

**Supplementary Table 1. Details of first and second primary cancers in Li Fraumeni Syndrome cohort where first cancer occurred ≤ 16 years.**

| Patient ID | Biological Sex | Age first cancer (years) | First cancer type | First cancer site | XRT for first cancer? | Second cancer type | Second cancer site | Second cancer in XRT field for first cancer? |
|---|---|---|---|---|---|---|---|---|
| 1 | M | 3.5 | fibrosarcoma | skull, right temporal | Yes | osteosarcoma | right fibula | no |
| 2 | M | 3.2 | embryonal rhabdosarcoma | right forearm | Yes | chondrosarcoma | rib, sternum, clavicle, associated joints | no |
| 3 | F | 2.6 | adrenocortical carcinoma | right adrenal | Yes | chondrosarcoma | left lower 5th and 6th ribs | yes |
| 4 | F | 1.1 | adrenocortical carcinoma | laterality not known | Yes | [1]smooth muscle tumour of uncertain malignant potential | right inguinal | yes |
| 5 | F | 1.4 | adrenocortical carcinoma | right adrenal | Yes | periosteal osteosarcoma ilium or chondroblastic osteosarcoma | ilium | yes |
| 6 | F | 9.6 | embryonal rhabdosarcoma | right pinna | Yes | breast phyllodes | right breast | no |
| 7 | F | 5.3 | choroid plexus carcinoma | left lateral ventricle | Yes | high-grade chondrosarcoma | right frontal region | yes |
| 8 | M | 0.4 | embryonal rhabdosarcoma with anaplasia, somatic translocation negative for PAX3/PAX7/FOXO1 | right chest wall | yes | high grade osteoblastic osteosarcoma | right 3rd rib | yes |

| | | | | | | | | |
|---|---|---|---|---|---|---|---|---|
| 9 | F | 4.3 | medulloblastoma | cerebellum | Yes | osteosarcoma | skull, occipito-parietal | yes |
| 10 | M | 1.7 | adrenocortical carcinoma | not known | Yes | rhabdomyosarcoma | retroperitoneal | yes |
| 11 | F | 0.7 | adrenocortical carcinoma | left adrenal | Yes | spindle cell sarcoma | left breast | yes |
| 12 | M | 1.4 | embryonal rhabdosarcoma with anaplasia | right lumbar paraspinal | Yes | not happen | not applicable | not applicable |
| 13 | M | 14.7 | osteosarcoma | right femur | Yes | not happen | not applicable | not applicable |
| 14 | M | 0.7 | embryonal rhabdomyosarcoma (pleiomorphic) | right middle ear | Yes | not happen | not applicable | not applicable |
| 15 | M | 2.6 | rhabdomyosarcoma | lung, laterality not known | Yes | not happen | not applicable | not applicable |
| 16 | F | 15.0 | adrenocortical carcinoma | left adrenal | Yes | not happen | not applicable | not applicable |
| 17 | M | 2.1 | embryonal rhabdomyosarcoma (pleiomorphic) | nasopharynx | Yes | not happen | not applicable | not applicable |
| 18 | M | 10.2 | astrocytoma (high-grade) | left hemisphere | Yes | not happen | not applicable | not applicable |
| 19 | F | 4.4 | adrenocortical carcinoma | right adrenal | Yes | not happen | not applicable | not applicable |

| | | | | | | | | |
|---|---|---|---|---|---|---|---|---|
| 20 | F | 1.0 | embryonal rhabdomyosarcoma | right leg | yes | not happen | not applicable | not applicable |
| 21 | F | 5.5 | rhabdomyosarcoma (primitive cell type, no alveolar pattern) | not known | Yes | not happen | not applicable | not applicable |
| 22 | F | 13.7 | renal cell carcinoma | laterality not known | No | liposarcoma | perineum | not applicable |
| 23 | M | 2.1 | adrenocortical carcinoma | right adrenal | No | astrocytoma, grade 2, IDH-1 mutation positive | left frontal lobe | not applicable |
| 24 | F | 0.8 | wilms | left | No | melanoma in situ | not known | not applicable |
| 25 | F | 13.3 | osteosarcoma | left lower limb | Yes | high-grade metaplastic cancer with rhabdomyosarcoma differentiation (carcinosarcoma) | breast | [2]yes |
| 26 | F | 15.1 | osteosarcoma | right fibula | No | osteosarcoma | right 7th rib | not applicable |
| 27 | F | 5.8 | adrenocortical carcinoma | right adrenal | No | spindle cell sarcoma | right wrist | not applicable |
| 28 | F | 1.7 | adrenocortical carcinoma | laterality not known | No | breast cancer x2. Both IDC grade 3 ERpos PRpos | bilateral breast | not applicable |
| 29 | F | 2.5 | adrenocortical carcinoma | laterality not known | No | Breast-atypical medullary | laterality not known | not applicable |
| 30 | F | 6.3 | rhabdomyosarcoma | tongue | No | breast IDC G3 ERpos PRneg HER2pos, and high grade DCIS | right | not applicable |

| | | | | | | | | |
|---|---|---|---|---|---|---|---|---|
| 31 | F | 1.2 | adrenocortical carcinoma | left adrenal | No | not happen | not applicable | not applicable |
| 32 | F | 1.2 | adrenocortical carcinoma | laterality not known | No | not happen | not applicable | not applicable |
| 33 | M | 3.3 | rhabdomyosarcoma | not known | No | not happen | not applicable | not applicable |
| 34 | M | 4.8 | rhabdomyosarcoma | right temporalis | No | astrocytoma x2. Both G2, IDH-1 mutation neg. | right frontal and left parietal | no |
| 35 | F | 1.9 | adrenocortical carcinoma | right adrenal | No | not happen | not applicable | not applicable |
| 36 | F | 2.8 | adrenocortical carcinoma | right adrenal | No | not happen | not applicable | not applicable |
| 37 | F | 4.2 | adrenocortical carcinoma | laterality not known | No | not happen | not applicable | not applicable |
| 38 | F | 11.3 | osteosarcoma | not known | No | not happen | not applicable | not applicable |
| 39 | M | 13.9 | osteosarcoma | not known | No | not happen | not applicable | not applicable |
| 40 | F | 0.0 | osteosarcoma | left lower femur | No | not happen | not applicable | not applicable |
| 41 | M | 13.7 | osteosarcoma | jaw | No | not happen | not applicable | not applicable |
| 42 | F | 7.4 | primitive neuroectodermal tumour | brain | No | not happen | not applicable | not applicable |
| 43 | M | 0.0 | rhabdomyosarcoma | not known | No | not happen | not applicable | not applicable |
| 44 | F | 9.7 | osteosarcoma | not known | No | not happen | not applicable | not applicable |
| 45 | F | 5.5 | triton tumour | left forearm | No | not happen | not applicable | not applicable |

| | | | | | | | | |
|---|---|---|---|---|---|---|---|---|
| 46 | F | 1.0 | adrenocortical carcinoma | laterality not known | No | not happen | not applicable | not applicable |
| 47 | F | 2.3 | adrenocortical carcinoma | right adrenal | No | not happen | not applicable | not applicable |

[1] Pleiomorphic leiomyosarcoma of abdomen developed 6 months later

[2]XRT was received to lung metastasis present at diagnosis of the osteosarcoma
